# Supplementary material for: Experimental investigation of the creep damage evolution of coal rock around gas extraction boreholes at different water contents
Source: PLoS One. 2023 Feb 16;18(2):e0278783. doi: 10.1371/journal.pone.0278783 (PMC9934311; doi:10.1371/journal.pone.0278783)
Supplement: Attachment — Submitted filename: Response to Reviewers101.docx [file pone.0278783.s002.docx]

Response to Reviewers

1. We note that several of your files are duplicated on your submission. Please remove any unnecessary or old files from your revision, and make sure that only those relevant to the current version of the manuscript are included.

Response: OK, I have removed the duplicates and updated the file list. Please check it.

2. Thank you for stating the following financial disclosure:“This work was supported by the National Natural Science Foundations of China, Study on mechanism and parameter optimization of carbon dioxide deep hole pre-split blasting in coal seam (Grant No. 51874234).”

Please state what role the funders took in the study. If the funders had no role, please state: "The funders had no role in study design, data collection and analysis, decision to publish, or preparation of the manuscript." If this statement is not correct you must amend it as needed. Please include this amended Role of Funder statement in your cover letter; we will change the online submission form on your behalf.

Response: The grant provided an important role for the thesis. I have submitted the appropriate instructions in the cover letter. Please check it.
